# Case Report: Brain tumor’s pitfalls: two cases of high-grade brain tumors mimicking autoimmune encephalitis with positive onconeuronal antibodies

**DOI:** 10.3389/fonc.2023.1254674

**Published:** 2023-08-24

**Authors:** Stefano Consoli, Fedele Dono, Giacomo Evangelista, Clarissa Corniello, Marco Onofrj, Astrid Thomas, Stefano L. Sensi

**Affiliations:** ^1^ Department of Neuroscience, Imaging and Clinical Science, “G. d’Annunzio” University of Chieti-Pescara, Chieti, Italy; ^2^ Epilepsy Center, “SS Annunziata” Hospital, Chieti, Italy; ^3^ Behavioral Neurology and Molecular Neurology Units, Center for Advanced Studies and Technology (CAST), University “G. d’Annunzio” of Chieti-Pescara, Chieti, Italy; ^4^ Institute for Advanced Biomedical Technologies, University of Chieti-Pescara, Chieti, Italy

**Keywords:** glioblastoma, autoimmune encephalitis, diagnosis, neuroimaging, epilepsy

## Abstract

**Background:**

Glioblastoma (GBM) is the most common primary brain tumor in adulthood. Initial diagnosis is generally based on clinical and MRI findings, which may be misinterpreted as other neurological pictures, including autoimmune encephalitis (AE). AE is a heterogeneous group of neuroinflammatory diseases due to the presence of auto-antibodies targeting antigens on neuronal synaptic or cell surface. In the present report, we describe two peculiar cases of GBM initially misdiagnosed as AE, focusing on the diagnostic pitfalls and the treatment strategies.

**Methods:**

We report the case of two patients with high-grade brain tumors, initially misdiagnosed and treated for AE. Clinical, laboratory, and neuroradiological data are discussed in terms of differential diagnosis between AE and GBM.

**Results:**

The presence of atypical brain MRI findings and the unresponsiveness to immunosuppressive treatment are major red flags in the differential diagnosis between AE and GBM. In these cases, a brain biopsy is necessary to confirm the diagnosis.

**Conclusions:**

Atypical brain tumor presentation causes a diagnostic and therapeutic delay. A positive onconeural autoantibodies result should always be interpreted cautiously, considering the possibility of a false-positive test. A brain biopsy is mandatory for a definite diagnosis.

## 1 Introduction

Glioblastomas (GBMs) are the most common primary brain tumors in adulthood, with a peak incidence between 65 and 75 years of age (1) and a median lifespan ranging from 8 to 14 months. According to tumor localization, GBM clinical manifestations spread from asymptomatic to severe neurological pictures. They may include neuropsychological changes, new-onset seizures, motor and/or sensory deficits, gate instability, cerebellar signs, and parkinsonism (2, 3). According to the 2021 CNS Classifications (4), the molecular characterization of primary brain tumors is critical for accurate diagnosis. Generally, GBMs are defined as IDH-wildtype tumors which may be associated with several further mutations, including telomerase reverse transcriptase (TERT) promoter mutations, epidermal growth factor receptor (EGFR) amplification, and concurrent gain of chromosome 7/loss of chromosome 10 [+7/-10] (5). Molecular changes are crucial for both therapeutic and prognostic purposes. It has been shown that temozolomide (TMZ) treatment is more effective in patients with peculiar molecular fingerprinting. In addition, some molecular findings, like unmethylated methylguanine-DNA methyltransferase (MGMT) and TERT gene promoter mutation, are generally related to poorer prognosis (5, 6).

Magnetic resonance imaging (MRI) of the brain is mandatory for GBM diagnosis (7). Typical brain MRI findings include: 1) hypointense to isointense lesions on T1-weighted sequences, 2) heterogeneous contrast enhancement uptake with a rim shape pattern indicative of necrosis, 3) hyperintense lesions on T2-weighted and fluid-attenuated inversion recovery (FLAIR) sequences associated with surrounding vasogenic edema (8). Moreover, advanced MRI techniques such as perfusion-weighted imaging (PWI) and magnetic resonance spectroscopy (MRS) could add further details. Indeed, PWI generally shows an increased relative cerebral blood volume, whereas MRS shows an increased choline and lactate pick with decreased N-acetyl-aspartate (9). Notwithstanding, MRI findings may sometimes be atypical and mimic various neurological conditions, including autoimmune encephalitis (AE) (10, 11).

AE is an antibody-mediated brain inflammatory process prompted by antibodies against intracellular or extracellular antigens (12). The AEs encompass a broad clinical spectrum that ranges from neurological and neuropsychiatric symptoms (e.g., changes in behavior or cognition, psychosis, abnormal movements, gate instability, aphasia, and depression) to subtle cognitive decline and seizures (13, 14). Diagnosis of AE is supported by several laboratory and neuroradiological findings (12, 15). Cerebrospinal fluid (CSF) analysis may highlight lymphocytic pleocytosis, increased proteins, or oligoclonal bands (OCBs) positivity, though patients with unremarkable findings have also been reported (15). Brain MRI generally shows T2/FLAIR hyperintense signal on the bilateral mesial temporal lobes or, less frequently, the lateral temporal lobe and the insula (16). However, heterogeneous patterns with multifocal brain lesions involving the cerebral cortex, the basal ganglia, or the brainstem have also been reported (17).

The differential diagnosis between GBM and AE may be challenging due to the possible unspecific clinical and neuroradiological findings which can be associated with both conditions. In the present report, we describe two peculiar cases of GBM initially misdiagnosed as AE, focusing on the diagnostic pitfalls and the treatment strategies.

## 2 Case presentation

### 2.1 Case 1

**Figure 1:**
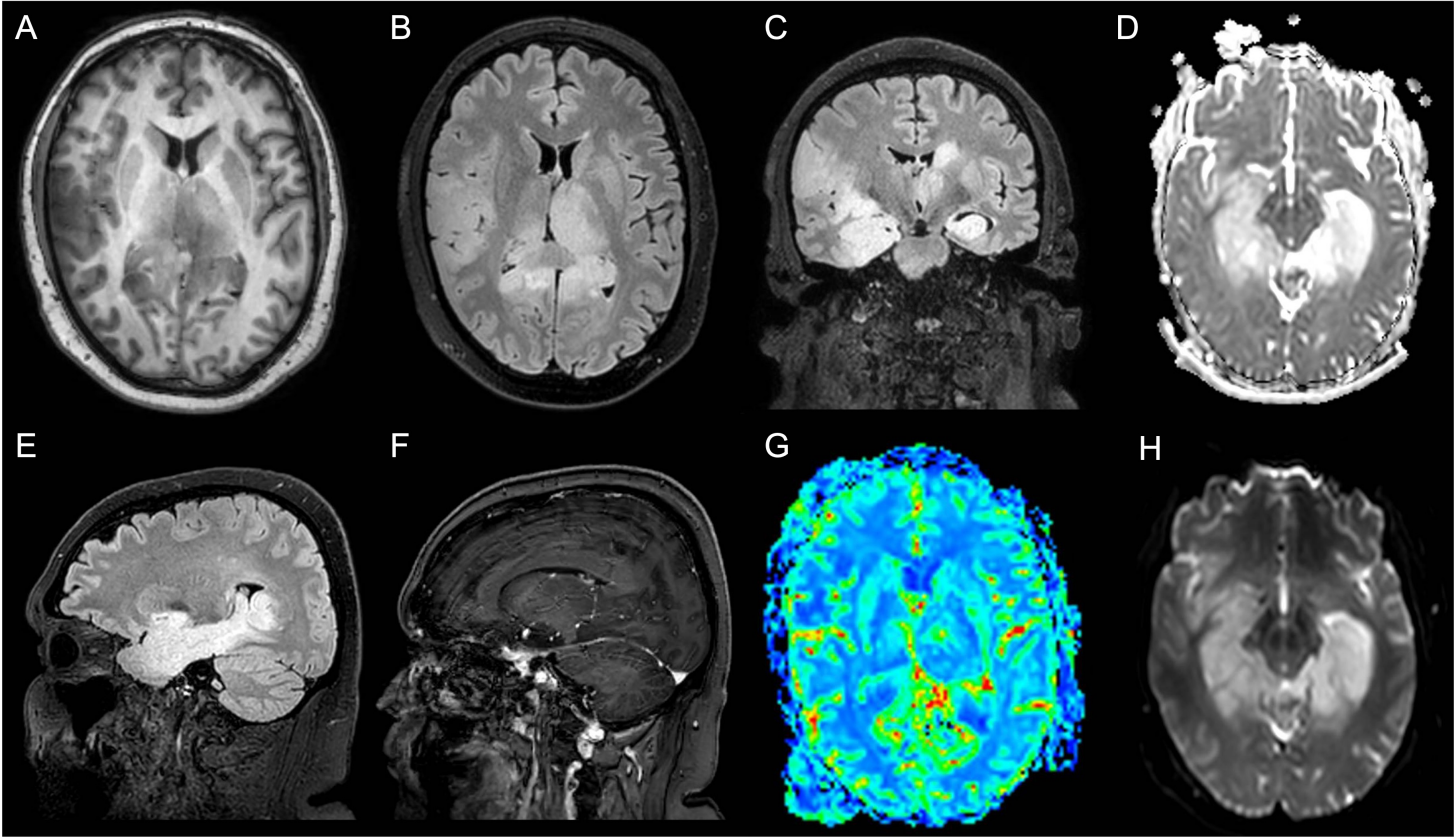
Patient 1 MRI scan of the brain shows diffuse cortico-subcortical T2 and Fluid Attenuated Inversion Recovery (FLAIR) images hyperintense lesions involving the bilateral hippocampal, the fusiform gyri, the right frontoparietal cortex, the left thalamus, and the right pulvinar. **(A)** Axial T1-weighted image; **(B)** Axial FLAIR image; **(C)** Coronal FLAIR image; **(D)** Axial apparent diffusion coefficient (ADC) image; **(E)** Sagittal FLAIR image; **(F)** Sagittal made of contrast positive T1-weighted image; **(G)** Axial perfusion weighted (PWI) image; **(H)** Axial diffusion weighted image (DWI).

A 40-year-old, right-handed man was admitted to the emergency room (ER) due to the recurrence of focal impaired awareness seizures with behavioral arrest. The patient’s past medical history revealed a subtle amnesic cognitive impairment with brief episodes of amnesia and behavioral disorders (i.e., agitation and crying spells) started about 3 months before the seizure onset, concomitantly to the administration of the second dose of the Sars-CoV2 vaccine (Comirnaty, Pfizer-BioNTech COVID-19 Vaccine). The patient underwent a brain MRI, which showed diffuse cortico-subcortical T2 and FLAIR hyperintense lesions involving the bilateral hippocampal and fusiform gyri, the right frontoparietal cortex, the left thalamus, and the right pulvinar (**Figures 1A–F**). In the suspicion of an AE, a lumbar puncture was performed, which was unremarkable. In addition, the autoimmune panel for surface and intracellular neuronal antibodies, executed on both CSF and serum, was negative. A possible post-SARS-CoV-2 vaccine acute disseminated encephalomyelitis (ADEM) was therefore diagnosed. Corticosteroid (methylprednisolone 1 g for 5 days) and anti-seizure (Levetiracetam 1500 mg/day) therapies were started, which led to a gradual improvement of the seizure frequency and the behavioral disturbance. However, a further neuropsychological evaluation revealed the persistence of cognitive deficits with executive functions and short- and long-term memory involvement.

After five months, the patient’s cognitive symptoms worsened. A new lumbar puncture showed no pathological findings. Nevertheless, the autoimmune autoantibodies panel for AE showed the positivity of anti-recoverin antibodies in the serum (titer 1:32) (**Table 1**). A brain MRI and MSR (**Figures 1G, H**) were performed, which revealed T2 and FLAIR diffuse hyperintense lesions with high signals in diffusion-weighted imaging (DWI) and increased Choline/N-acetyl-aspartate ratio (Ch/Naa>2). Thus, a neuroradiological diagnosis of gliomatosis cerebri was made. A stereotactic brain biopsy and subsequent neuropathological evaluations were performed, resulting in a glioma tumor isocitrate dehydrogenase 1 (IDH-1) and 2 (IDH-2) wild type, glial fibrillary acidic protein (GFAP) and oligodendrocyte transcription factor 2 (Olig2) positive. The proliferation index, indexed by Mindbomb Homolog-1 (MIB-1) antibody, was 10% associated with an area of necrosis. Therefore, the molecular and histological characteristics resulted in a diagnosis of glioblastoma (grade 4, WHO 2021). Due to the MRI characteristics of the lesion, a neurosurgical approach was ruled out in favor of combined treatment with radiotherapy (2 Gy per day, total 60 Gy) and chemotherapy (temozolomide, 75 mg/m^2^ during radiotherapy followed by 6 cycles of 200 mg/m^2^ for 5 days each 28-day cycle), in line with the Stupp Protocol.

**Table 1:** Patients’ cerebrospinal fluid (CSF) and serum analysis characteristics.

Cerebrospinal fluid analysis				
*Patient 1*	*Cytochemical examination*	*Result*	*Autoimmune panel*	*Titer*
	*Appearance*	Clear	*Anti Ca^2+^ Channel Ab*	Negative
	*White cells*	3 cells/mm^3^	*Aanti VGCK Ab*	Negative
	*Glucose*	60.4 mg/dl	*Anti GLUR3 Ab*	Negative
	*Proteins*	31.8 mg/dl	*Anti AMPAR1,2 Ab*	Negative
	*Microbiological panel*		*Anti CASPR2 Ab*	Negative
	*HSV-1*	Negative	*Anti LGI1 Ab*	Negative
	*HSV-2*	Negative	*Anti NMDAR Ab*	Negative
	*HHV-6*	Negative	*Anti GABAR Ab*	Negative
	*HHV-7*	Negative	*Anti GAD65 Ab*	Negative
	*HHV-8*	Negative	*Anti MOG Ab*	Negative
	*CMV*	Negative	*Anti AQP4 Ab*	Negative
	*EBV*	Negative	*Oligoclonal bands*	Negative
	*VZV*	Negative		
*Patient 2*	*Cytochemical examination*	*Result*	*Autoimmune panel*	*Titer*
	*Appearance*	Clear	*Anti Ca^2+^ Channel Ab*	Negative
	*White cells*	1 cells/mm^3^	*Aanti VGCK Ab*	Negative
	*Glucose*	54 mg/dl	*Anti GLUR3 Ab*	**1:2**
	*Proteins*	26 mg/dl	*Anti AMPAR1,2 Ab*	Negative
	*Microbiological panel*		*Anti CASPR2 Ab*	Negative
	*HSV-1*	Negative	*Anti LGI1 Ab*	Negative
	*HSV-2*	Negative	*Anti NMDAR Ab*	Negative
	*HHV-6*	Negative	*Anti GABAR Ab*	Negative
	*HHV-7*	Negative	*Anti GAD65 Ab*	Negative
	*HHV-8*	Negative	*Anti MOG Ab*	Negative
	*CMV*	Negative	*Anti AQP4 Ab*	Negative
	*EBV*	Negative	*Oligoclonal bands*	Negative
	*VZV*	Negative		

Serum analysis				
*Patient 1*	*Autoimmune panel*	*Titer*		
	*Anti Ca^2+^ Channel Ab*	Negative	Anti-Ma1 Ab	Negative
	*Anti VGCK Ab*	Negative	Anti-Ma2/Ta Ab	Negative
	*Anti GLUR3 Ab*	Negative	Anti-CV2/CRMP5 Ab	Negative
	*Anti AMPAR1,2 Ab*	Negative	Anti-Hu Ab	Negative
	*Anti CASPR2 Ab*	Negative	Anti-Ri p54 Ab	Negative
	*Anti LGI1 Ab*	Negative	Anti-Yo Ab	Negative
	*Anti NMDAR Ab*	Negative	Anti-recoverin Ab	***1:32***
	*Anti GABAR Ab*	Negative	Anti-amphiphysin Ab	Negative
	*Anti GAD65 Ab*	Negative	Anti-SOX1 Ab	Negative
	*ANA*	Negative	Anti-Zic4 Ab	Negative
	*ENA*	Negative	Anti-titin Ab	Negative
	*Antiphospholipid antibodies*	Negative	Anti-Tr Ab	Negative
*Patient 2*	*Autoimmune panel*	*Titer*		
	*Anti Ca^2+^ Channel Ab*	Negative	Anti-Ma1 Ab	Negative
	*Anti VGCK Ab*	Negative	Anti-Ma2/Ta Ab	Negative
	*Anti GLUR3 Ab*	**1:200**	Anti-CV2/CRMP5 Ab	Negative
	*Anti AMPAR1,2 Ab*	Negative	Anti-Hu Ab	Negative
	*Anti CASPR2 Ab*	Negative	Anti-Ri p54 Ab	Negative
	*Anti LGI1 Ab*	Negative	Anti-Yo Ab	Negative
	*Anti NMDAR Ab*	Negative	Anti-recoverin Ab	Negative
	*Anti GABAR Ab*	Negative	Anti-amphiphysin Ab	Negative
	*Anti GAD65 Ab*	Negative	Anti-SOX1 Ab	Negative
	*ANA*	Negative	Anti-Zic4 Ab	Negative
	*ENA*	Negative	Anti-titin Ab	Negative
	*Antiphospholipid antibodies*	Negative	Anti-Tr Ab	Negative

AMPAR, alpha-amino-3-hydroxy-5-methyl-4-isoxazolepropionic acid receptor; ANA, antinuclear antibody; AQP4, aquaporin-4; CASPR2, anti-contactin-associated protein-like 2; CMV, cytomegalovirus; CV2/CRMP5, collapsin response mediator protein; EBV, Epstein-Barr virus; GABAR, anti-y-aminobutyric acid-beta-receptor 1; ENA, extractable nuclear antigen; GAD65, glutamic acid decarboxylase 65; GLUR3, glutamate receptor 3; HSV, herpes simplex virus; HHV, human herpes virus; LGI1, leucine-rich glioma inactivated 1; MOG, myelin oligodendrocyte glycoprotein; NMDAR, N-methyl-D-aspartate receptor; SOX1, superoxide dismutase 1; Tr, thyrotropin; VGCK, voltage-gated potassium channel; VZV, varicella-zoster virus; Zic4, zinc finger protein. Bold numbers are used to identify pathological findings.

At the 1-year visit follow-up, the patient was autonomous in daily activities but could not resume work (Karnofsky score: 70%). The patient did not complain of any severe adverse effects related to CT/RT regimens. However, persistent short-term memory deficits and depressive symptoms were reported. Furthermore, the patient referred recurrent daily episodes of epigastric sensation and dejà-vu, which were otherwise interpreted as focal aware seizure. A concomitant EEG showed sporadic diphasic high-amplitude sharp waves on the left anterior temporal lobe regions. A treatment with Lamotrigine 200 mg/die was then introduced with moderate benefit on the depressive symptoms and a reduction of>50% of seizure frequency. The MRI scan of the brain showed no modification of the neuroradiological picture.

**Figure 2:**
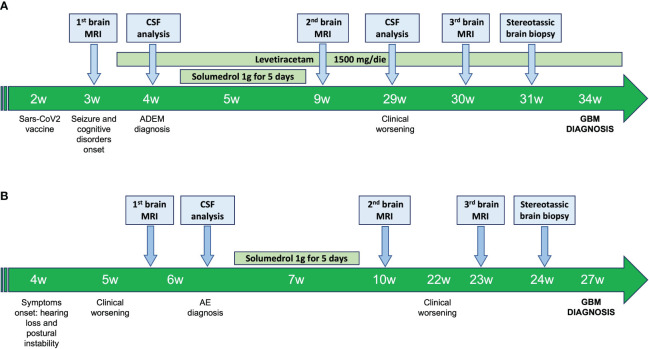
Patient 1 **(A)** and patient 2 **(B)** time-line events. ADEM, Acute disseminated encephalomyelitis; AE, Autoimmune encephalitis; CSF, Cerebrospinal Fluid; GBM, Glioblastoma; MRI, Magnetic Resonance Image of the brain.

The patient’s time-line events are summarized in **Figure 2A**.

### 2.2 Case 2

A 35 years-old, right-handed man was admitted to the ER for persistent (i.e., 30 days) and slowly progressive hearing loss and postural instability. The patient’s past medical history was unremarkable. At the admission, the patient showed a right sensorineural hearing loss and nystagmus in all directions of gaze, associated with slight weakness of the right lower limb.

**Figure 3:**
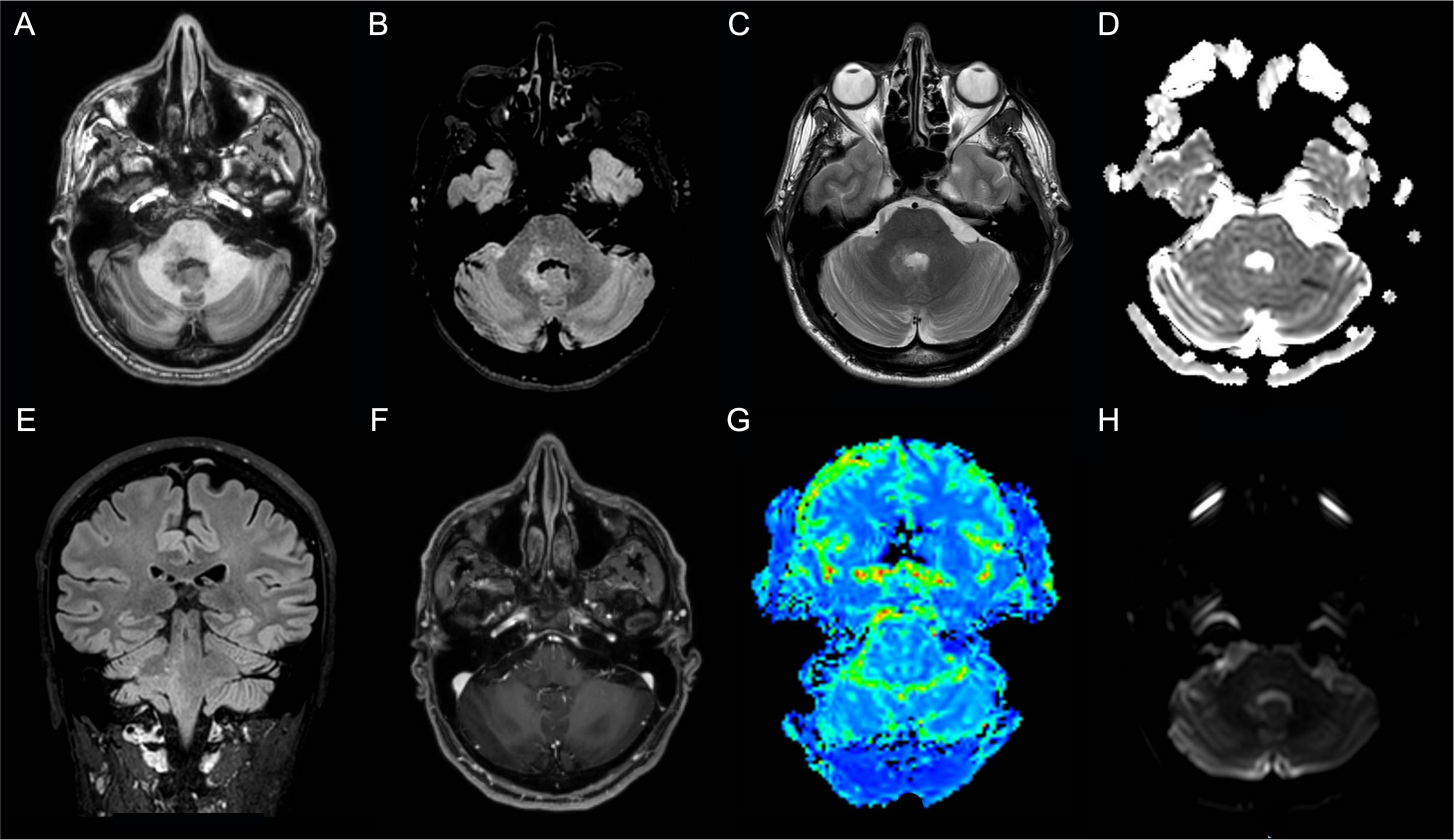
Patient 2 MRI scans of the brain shows T2-weighted and Fluid Attenuated Inversion Recovery (FLAIR) images hyperintense blurred lesion on the right pontine-bulbar portion and the ipsilateral superior and middle cerebellar peduncles **(A)** Axial T1-weighted image; **(B)** Axial FLAIR image; **(C)** Axial T2-weighted image; **(D)** Axial apparent diffusion coefficient (ADC) image; **(E)** Sagittal FLAIR image; **(F)** Axial made of contrast positive T1-weighted image; **(G)** Axial perfusion weighted (PWI) image; **(H)**) Axial diffusion weighted image (DWI).

Thus, the patient underwent a brain MRI, which showed a T2/FLAIR hyperintense blurred lesion on the right pontine-bulbar portion and the ipsilateral superior and middle cerebellar peduncles. The PWI, as well as the MRS, were unremarkable (**Figures 3A–H**). In the suspicion of rhombencephalitis, a lumbar puncture was performed, which showed normal cell and protein levels as well as no bacterial (i.e., Listeria, Tuberculosis, and Mycoplasma) and viral (e.g., Herpes simplex virus 1 and 2, and Cytomegalovirus) infection. However, the autoimmune panel for surface and intracellular neuronal antibodies revealed positivity for anti-GluR3 antibodies both in the CSF (titer 1:2) and in the serum (titer 1:200) (**Table 1**). On the contrary, serum Myelin Oligodendrocyte Glycoprotein (MOG) and Aquaporin 4 (AQP4) antibodies resulted in normal. Thus, the patient was treated with methylprednisolone (1 g/day for 5 days) followed by oral prednisolone at 50 mg/day, and a moderate improvement of neurological symptoms was observed.

After 3 months, a worsening of the clinical picture occurred. In particular, the patient developed right hemiparesis and appeared more confused and irritable. A brain lesion biopsy revealed a high-grade infra-tentorium IDH-1 and IDH-2 wild-type glial tumor, GFAP and Olig2 positive and histone H3‐K27M mutation-negative (glioblastoma, grade 4 WHO 2021). The proliferation index (MIB-1) was 35%.

A surgical approach and adjuvant radiotherapy (2 Gy per day, total 60 Gy) and chemotherapy (temozolomide, 75 mg/m^2^ during radiotherapy followed by 6 cycles of 200 mg/m^2^ for 5 days each 28-day cycle) in line with the Stupp Protocol were then attempted. However, due to a severe reduction in platelet count, chemotherapy was discontinued after six weeks.

The last follow-up was performed one year after surgery. The patient showed persistent right sensorineural hypoacusia, conjugate rightward gaze paralysis, and diplopia with inexhaustible nystagmus in leftward lateral gaze. In addition, personal self-sufficiency was severely affected by walking difficulties and right-side weakness (Karnofsky score: 60%). The MRI scan of the brain showed no modification of the neuroradiological picture.

The patient’s time-line events are summarized in **Figure 2B**.

## 3 Discussion

GBM’s presentation may be very heterogeneous in terms of imaging and clinical findings, sometimes mimicking other neurological conditions such as AE (18).

In the two cases we reported, patients presented atypical neuroradiological and clinical features that did not immediately lead to a brain tumor diagnosis. In the first case, a diffuse cortico-subcortical involvement, as demonstrated by brain MRI scans, strongly supported the diagnosis of inflammatory encephalitis (11). Indeed, to date, less than 2% of individuals with malignant gliomas have been documented to have multicentric GBMs showing a pattern of *gliomatosis cerebri* (19). In the second case, the specific localization of the brain lesion raised some doubts regarding its actual etiology. Adults rarely develop primary infra-tentorial glioblastoma, and cerebropontine angle (CPA) location is estimated to be even rarer (incidence range from 1.5% to 4.1%) (20, 21). Unfortunately, in both cases, the employment of advanced MRI techniques (i.e., PWI and MRS) failed to help elucidate the specific etiology.

From a clinical point of view, both patients presented several neurological symptoms with a subacute onset mimicking the presentation of an AE. In particular, the new-onset seizures associated with subacute neuropsychological deficits observed in the first patient agreed with the AE diagnosis according to Grauss’ criteria (12). On the other hand, the signs of cerebellar and pontine-bulbar involvement observed in the second patient raised the suspicion of inflammatory rhombencephalitis (22, 23). However, laboratory tests performed on serum and CSF partially confirmed the diagnosis of AE. Although both patients showed serum and/or CSF positivity for onconeural autoantibodies, there was a discrepancy between the specific antibodies-related syndrome and the actual clinical features. Anti-amphiphysin antibodies are typically associated with stiff-person syndrome (SPS) (i.e., a neurological syndrome characterized by axial rigidity, muscle stiffness, and startle reflex) (24, 25) whereas anti-GluR3 antibodies are generally observed in Rasmussen encephalitis, untreatable epilepsy and, less frequently, rhombencephalitis (26, 27). Thus, while the second patient showed some clinical symptoms which could fit with an anti-GluR3 encephalitis diagnosis, the first patient did not show any manifestations which could even raise suspicion of SPS.

However, in the first patient, some anamnestic data supported the hypothesis of an inflammatory brain process. Indeed, the close correlation between the SARS-CoV-2 vaccine administration and the onset of the symptoms raised suspicion of post-vaccine acute disseminated encephalomyelitis (ADEM) (28). ADEM usually affects younger patients, but several cases during adulthood have also been described specifically in the context of SARS-CoV-2 vaccination (29).

In both cases, immunosuppressive therapy with high-dose corticosteroids was started, with mild-to-moderate effects on neurological symptoms. However, due to the atypical radiological presentation and the successive worsening of the clinical picture, we sought to perform a stereotactic brain biopsy by which a histological and molecular diagnosis of glioblastoma (GBM grade 4, WHO 2021) was ascertained. It has to be pointed out that though the use of corticosteroids is allowed in patients suffering from GBM due to the well-known anti-edema effects (30), more aggressive immunosuppressive treatment (e.g., azathioprine, cyclophosphamide, anti-CD20 monoclonal antibodies), commonly used to treat AE refractory to corticosteroids, should be avoided given the possible detrimental effects on tumor progression.

However, in our cases, the brain biopsy led to the correct diagnosis, allowing the most appropriate therapies and avoiding invasive and potentially harmful treatments.

## 4 Conclusion

Glioblastomas (GBMs) could present with similar clinical and radiological findings that can be seen in autoimmune encephalitis (AE), leading to diagnostic and treatment delays. A positive onconeural autoantibodies result should always be interpreted with caution, taking into account the possibility of a false-positive test. A biopsy should be performed before starting a potentially harmful therapy, especially in case of unusual symptoms and radiological features. Neurologists should always consider the possibility of an atypical presentation of a relatively common disease, keeping in mind the heterogeneous clinical and radiological behavior of glioblastoma.

## Data availability statement

The raw data supporting the conclusions of this article will be made available by the authors, without undue reservation.

## Ethics statement

Ethical approval was not provided for this study on human participants because written informed consent was obtained from the patient to publish this case report and any accompanying images. The paper is exempt from ethical committee approval because it is not necessary for the publication of the case report. We confirm that we have read the Journal’s position on issues involved in ethical publication and affirm that this report is consistent with those guidelines. The patients/participants provided their written informed consent to participate in this study. Written informed consent was obtained from the patient to publish this case report and any accompanying images.

## Author contributions

SC: Conceptualization, Data curation, Writing – original draft. FD: Conceptualization, Data curation, Formal Analysis, Investigation, Methodology, Project administration, Resources, Supervision, Validation, Visualization, Writing – original draft, Writing – review & editing. GE: Data curation, Investigation, Writing – review & editing, Data curation, Investigation, Writing – review & editing. CC: Investigation, Writing – review & editing. MO: Resources, Supervision, Validation, Visualization, Writing – review & editing. AT: Supervision, Validation, Visualization, Writing – review & editing. SS: Data curation, Supervision, Validation, Visualization, Writing – review & editing.
